# Physical function is associated with cognitive status, brain amyloid‐beta deposition, and blood biomarkers in Chinese Han population

**DOI:** 10.1111/cns.14921

**Published:** 2024-08-18

**Authors:** Yuhuai Guo, Lin Huang, Junliang Kuang, Tao Sun, Xiaoyan Zhang, Haili Tian, Fang Xie, Tianlu Chen, Qihao Guo

**Affiliations:** ^1^ Department of Gerontology Shanghai Sixth People's Hospital Affiliated to Shanghai Jiao Tong University School of Medicine Shanghai China; ^2^ Center for Translational Medicine and Shanghai Key Laboratory of Diabetes Mellitus Shanghai Sixth People's Hospital Affiliated to Shanghai Jiao Tong University School of Medicine Shanghai China; ^3^ School of Exercise and Health Shanghai University of Sport Shanghai China; ^4^ PET Center, Huashan Hospital Fudan University Shanghai China

**Keywords:** Alzheimer's disease, cognitive function, gait, grip strength, physical function

## Abstract

**Background:**

The physical function of elderly individuals reflects whether they have had a history of regular physical activity over the long term. Such indicators have been found to have a certain connection with cognitive function these years. However, there is limited research that associates it with mechanisms such as cerebral Aβ deposition. We aim to investigate this relationship and unveil the underlying mechanisms.

**Method:**

Physical function and cognition data of 4189 participants were obtained from the Chinese preclinical Alzheimer's disease study. Participants were divided into six groups according to disease severity. Among them, 1048 participants underwent the positron emission tomography‐computed tomography (PET‐CT) and plasma biomarker test. Grip strength and gait were combined into a score indicating physical function. Multiple linear regression models and logistic regression models were mainly used to conduct the analysis.

**Results:**

There was a significant positive correlation between physical function and cognitive function (*R* = 0.48, *p* < 0.001), independent of sex, age, apolipoprotein E‐ε4 genotype, and disease stages (*p* < 0.001). Physical function was effective in distinguishing individuals with cognitive impairment from those without (AUC = 0.835). Physical function was negatively associated with brain Aβ deposition (*p* = 0.008) and brain Aβ had an intermediary effect (*p* < 0.01) on the association between physical function and cognition in women. This association was mainly evident in the lateral parietal, lateral temporal, posterior cingulate, frontal, occipital, and precuneus regions. Physical function was negatively associated with plasma neurofilament light‐chain (Nfl) level (*p* < 0.001).

**Conclusions:**

Physical function is strongly associated with cognitive function in the Chinese elderly, and brain Aβ deposition partly mediates the linkage in women. Plasma Nfl can be used as a potential target for exercise intervention in cognitive function. Improving physical function will contribute to the alleviation of cognition decline.

## Graphical abstract

The physical function represented by grip strength and gait is significantly associated with cognitive function, and this relationship is not influenced by gender, age, genetic factors, or dementia severity. This connection allows us to distinguish whether a person's cognition is impaired based on their physical abilities. The brain Aβ deposition serves as an intermediate factor that partially mediates the relationship between physical function and cognitive function, and this mediating effect is particularly pronounced in the female population.

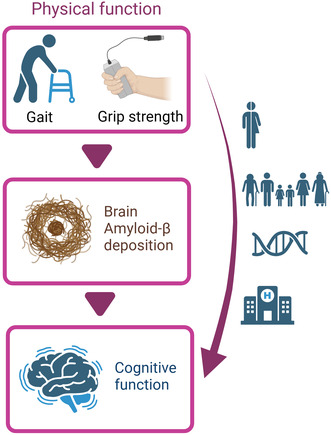

## 1 INTRODUCTION

Cognition decline is a common accompanying symptom of aging. However, the severe cognitive decline associated with Alzheimer's disease (AD) inflicts immeasurable suffering upon older adults and their families.^1^ Moreover, AD imposes a significant burden on the economy and society as a whole.^2^, ^3^ In 2022, the estimated healthcare costs associated with AD treatment were $321 billion, with costs projected to exceed $1 trillion by 2050.^3^ The onset of AD is not a sudden occurrence. Over time, the patient's memory and cognitive function gradually deteriorate, with the rate of decline tending to accelerate.^4^, ^5^ Hence, early diagnosis and management of AD have become increasingly crucial and receive significant attention.^6^ The investigation of blood and cerebrospinal fluid biomarkers has emerged as a key area of research in early AD detection.^7^, ^8^ By understanding the alterations in these biomarkers, it becomes possible to gain insights into the underlying mechanisms and develop more precise diagnostic tools and treatment strategies for AD. Numerous factors influence the development of AD, including age, sex, APOE alleles, family history, comorbidities, physical activity, healthy diet, and other lifestyle habits, and all of these factors are closely associated with the risk and progression of AD.^9^

Sarcopenia is a progressive and generalized skeletal muscle disorder involving the accelerated loss of muscle mass and function, resulting in increased adverse outcomes including falls, functional decline, frailty, and higher mortality.^10^ Both weakness defined by low grip strength and slowness defined by low usual gait speed are important characteristics of sarcopenia, receiving unanimous recognition as defining features of sarcopenia by an international panel of experts in the Sarcopenia Definition and Outcomes Consortium (SDOC).^11^ The combination of grip strength and gait is the most widely recognized and applied reliable phenotypic indicator in the elderly,^12^ encompassing both upper and lower limb physical function. It is not only limited to sarcopenia but is also used in the assessment of postoperative complications, hematological malignancies, cardiovascular, and other diseases.^13^, ^14^, ^15^ The decline in physical function, which is commonly observed in the elderly population, is closely associated with age‐related diseases. In this study, we utilized the maximum grip strength test and timed up and go test (TUGT) to assess these two aspects of physical function, given their easily obtainable, objectivity, and minimal time requirements. Subsequently, we employed statistical methods to integrate these measures into a comprehensive index for analysis.

In recent years, researchers have had a growing interest in exploring the relationship between physical function and cognitive decline‐related conditions.^16^, ^17^, ^18^ Some studies have suggested that the decline in physical function among older adults may precede the onset of cognitive decline, serving as a prodromal state.^19^, ^20^ Physical exercise has been widely recognized as a cognitive intervention strategy,^21^, ^22^, ^23^ and therefore, highlighting the need for investigation of the link between physical function and cognitive function. How does the improvement of physical function relate to cognitive function, given its reflection on long‐term exercise habits and interventions? Can physical function serve as a predictor of cognitive impairment and, if so, how does it contribute as a precursor factor in predicting cognitive decline? Up to now, this field of research remains relatively understudied, with limited research on the correlation between physical function and cognitive function. Studies in this field have often involved small sample sizes and have not been validated by pathophysiological biomarkers. Here, we performed a nested cross‐sectional analysis involving a total of 4189 individuals from the Chinese preclinical Alzheimer's disease study (C‐PAS), which was recruited from the Memory Clinic of Shanghai Sixth People's Hospital affiliated with Shanghai Jiao Tong University School of Medicine, and Cognitive Disorder Friendly Communities in Shanghai. Our aim is to investigate the association between cognitive function and physical function in Chinese older adults and to provide insights into the early diagnosis and intervention of AD.

## 2 SUBJECT AND METHODS

### 2.1 Study cohort and sample collection

A total of 4189 participants were included from the cohort of Chinese preclinical Alzheimer's disease study (C‐PAS). Written informed consent was obtained at the time of enrollment which was approved by the Institutional Ethics Reviewing Board of Shanghai Sixth People's Hospital affiliated to Shanghai Jiao Tong University School of Medicine. Complete information on C‐PAS study including inclusion and exclusion criteria, sample collection protocol, and clinical marker extraction (demographics, apolipoprotein E‐ε4 genotype (APOE‐4), neuropsychological test scores, diagnostic and grouping criteria, and plasma and positron emission tomography (PET) imaging markers) can be found at our previous publication.^24^ For the grip strength test, participants were instructed to stand with their arms hanging naturally by their sides. Each hand was tested three times using a dynamometer, and the highest value recorded was used as the test result. We conducted the timed up and go test following the standards specified by the American Physical Therapy Association (APTA).^25^ The study was reviewed and approved by the Ethics Committee of Shanghai Jiao Tong University Affiliated Sixth People's Hospital. It was performed in accordance with the principles of the Declaration of Helsinki (approval number 2019‐041). All participants provided written informed consent to participate in the study.

### 2.2 Study design and enrollment

The overall study design and the number of enrollments covered in this report are shown in Figure S1. Among the 4189 participants who were screened, 4053 participants were eventually included in the analysis. We excluded cases with missing values. The major grouping was based on the stratification of cognitive functions. (1) Normal cognition (NC): normal subjective sensory cognition; had no objective cognitive impairment that met objectively defined subtle cognitive decline (Obj‐SCD) or mild cognitive impairment (MCI) criteria. (2) Subjective cognitive decline (SCD): self‐experienced persistent cognition capacity decline impairment and associated worries with a course less than 5 years; and without objective cognitive impairment meeting the Obj‐SCD or MCI criteria. (3) MCI: Had an impaired score on both measures within at least one cognitive domain or had one impaired score in each of the three cognitive domains.^26^ (4) AD: Determined based on clinical diagnosis, meeting the National Institute on Aging–Alzheimer's Association (NIA‐AA) 2011 diagnostic criteria for probable AD dementia.^27^ We further categorized AD patients into three groups: mild AD (AD1, CDR = 1), moderate AD (AD2, CDR = 2), and severe AD (AD3, CDR = 3), based on the clinical dementia rating (CDR) criteria.^28^ The CDR is derived from a semi‐structured interview with the patient and an appropriate informant and rates impairment in each of six cognitive categories (memory, orientation, judgment and problem‐solving, community affairs, home and hobbies, and personal care) on a 5‐point scale in which none = 0, questionable = 0.5, mild = 1, moderate 2, and severe = 3.

### 2.3 Statistical analysis

All the data analysis was conducted using R (V3.5.1). All continuous variables included in the study were tested for normality using the Shapiro–Wilk test (Table S5). Since the majority of variables were not normally distributed, we used the Kruskal–Wallis test to compare differences in continuous variables between groups and the Chi‐square test to analyze categorical variables. Spearman correlation and partial correlation were used for correlation analysis. The first principal component (PC1) of principal component analysis (PCA) was employed as the representative variable for physical function, which integrated two indicators of physical function, the average grip strength of both hands (muscle strength), and the negative scores of the TUGT (balance and coordination). We used scatter plots to fit trend lines.

### 2.4 Identify confounders

To identify potential confounding factors that may influence the relationship between physical function and cognitive function, we used the Addenbrooke's cognitive examination (ACE‐III) score as the dependent variable and physical function as the independent variable to build a linear regression model. ACE and its related versions show better performance than mini‐mental state examination (MMSE) and Montreal cognitive assessment (MoCA) in detecting mild cognitive impairment in different neurological disorders.^29^, ^30^ ACE has a wider score range, with a total score of 0–100, making it easier to discern all cognitive function states.^31^ We then added a variable of interest as a covariate to the model and subtracted the coefficient of physical function in the adjusted model from the coefficient in the original model. We performed Bootstrap resampling (1000 times) to calculate the confidence interval of the coefficient difference. If the confidence interval did not cross zero, it may have a significant confounding effect. We applied this method to every variable of interest, one by one, to identify confounding factors that had a significant impact on the relationship between physical function and cognitive function.

### 2.5 Mediation effect analysis

We employed a similar Bootstrap method for conducting mediation analysis, calculating the significance of the mediating effects and the proportion of mediation in the total effect. Mediation analysis was utilized to investigate whether brain Aβ deposition mediates the relationship between physical function and cognition. All mediation analyses were adjusted for the confounding factors identified in the previous step.

### 2.6 Classification effect evaluation

When plotting the receiver operating characteristic curve (ROC) curves to differentiate different diagnostic groups based on physical function, we employed logistic regression and multinomial logistic regression models. The dataset was randomly split into training and testing sets using a non‐replacement method, with a 7:3 ratio for training and testing, respectively. Participants diagnosed as NC or SCD were classified into the non‐CI group (no cognitive impairment), while those diagnosed with MCI and AD were classified into the CI group (cognitive impairment). Physical function was used as the independent variable, and the grouping variables were used as the outcome variables. The best cutoff value for distinguishing high and low physical function was obtained using the Youden's Index method.

### 2.7 Sensitivity analysis

In the sensitivity analysis, we employed another method (coefficient‐weighted standardization) to integrate the TUGT score and grip strength. This method involved using TUGT and grip strength as independent variables and the ACE‐III score as the dependent variable in a multiple linear regression model. The coefficients of TUGT and grip strength in the equation were obtained, and the weights were calculated by dividing each coefficient by the sum of the two coefficients. The weighted values of TUGT and grip strength were then summed to obtain the composite measure of physical function.

## 3 RESULTS

### 3.1 Baseline characteristics

In total, the analysis incorporated 4189 participants, comprising 38.1% men and 61.9% women, with an average age of 67.9 ± 11.5 years old. Among the 4189 participants, 1253 (30.9%) were NC, 507 (12.5%) were SCD, 845 (20.8%) were MCI, 556 (13.7%) were mild AD, 608 (15.0%) were moderate AD, and 284 (7.0%) were severe AD. We observed that the severity of AD progression was more pronounced among those who were older (*p* < 0.001) or had lower levels of education (*p* < 0.001) (Table 1). Significant differences were also observed in body mass index (BMI) (*p* < 0.001), waist circumference (*p* < 0.05), smoking history (*p* < 0.05), and family history (*p* < 0.05) across different groups. Physical function (*p* < 0.001), grip strength (*p* < 0.001), and walking test scores (*p* < 0.001) showed a decline with the increase in AD progression.

**TABLE 1:** Demographic and clinical characteristics of study participants (*n* = 4189).

	ALL (*n* = 4189)	NC (*n* = 1253)	SCD (*n* = 507)	MCI (*n* = 845)	AD1 (*n* = 556)	AD2 (*n* = 608)	AD3 (*n* = 284)	*p* value
Age (year)	67.9 ± 11.5	61.8 ± 12.6	64.5 ± 8.1	68.9 ± 9.4	72.8 ± 8.8	73.6 ± 9.4	75.1 ± 10.1	<0.001***
Sex (M/F)	1597/2592	455/798	149/358	333/512	235/321	259/349	107/177	<0.001***
Education (year)	11.3 ± 4.2	12.8 ± 3.7	11.8 ± 3.3	11.2 ± 3.7	10.1 ± 4.7	10.1 ± 4.4	8.3 ± 4.9	<0.001***
Marriage (Y/N)^a^	2161/351	825/131	376/58	457/74	213/37	189/39	53/8	0.881
BMI (kg/m^2^)	23.3 ± 3.3	23.5 ± 3.1	23.8 ± 3.5	23.6 ± 3.4	22.9 ± 3.3	22.7 ± 3.5	22.8 ± 3.4	<0.001***
Wasit (cm)	85.3 ± 9.6	84.7 ± 9.9	84.7 ± 9.1	86.1 ± 9.6	86.5 ± 9.2	85.9 ± 9.6	86.6 ± 9.7	0.038**
MAP (mmHg)	96.6 ± 11.9	95.1 ± 12.7	95.7 ± 10.6	99.1 ± 10.2	98.8 ± 13.5	97.3 ± 11.2	100.7 ± 10.6	0.079*
Smoke (Y/N)	565/3308	159/1012	51/423	114/674	85/429	97/465	42/212	0.017**
Drink (Y/N)	671/3197	213/954	74/402	133/655	93/421	105/456	39/212	0.514
Family history (Y/N)	1562/1134	536/415	219/174	308/243	193/117	195/128	72/29	0.015**
Medication history (Y/N)	473/3727	33/1221	19/488	63/783	103/453	136/477	87/198	<0.001***
PET‐CT^d^	*n* = 1048, 25%	*n* = 363, 29%	*n* = 213, 42%	*n* = 232, 27%	*n* = 105, 19%	*n* = 87, 14%	*n* = 18, 6%	<0.001***
Aβ (+/−)	416/590	91/272	68/145	94/138	75/18	72/15	16/2	<0.001***
APOEe4 (+/−)	305/788	79/306	41/177	70/170	52/61	48/42	7/12	<0.001***
MMSE	22.9 ± 7	27.8 ± 2.9	27.3 ± 2.3	26.3 ± 2.2	21.2 ± 2.1	14.8 ± 2.8	6.4 ± 2.4	<0.001***
MoCA‐B	19.8 ± 7.1	25.4 ± 3.7	24.1 ± 3.6	20.6 ± 3.7	14.8 ± 3.9	10.3 ± 4	4.7 ± 2.5	<0.001***
ACE‐III	61.4 ± 21.8	80.5 ± 11.5	77.8 ± 8.8	69.0 ± 10.2	55.9 ± 10.4	40.6 ± 10.7	20.0 ± 10.4	<0.001***
Physical function^b^	0.0 ± 1.1	0.5 ± 0.9	0.4 ± 0.8	0.0 ± 0.9	−0.3 ± 1.1	−0.7 ± 1.3	−1.3 ± 1.3	<0.001***
Grip strength (kg)^c^	22.0 ± 8.5	24.7 ± 8.4	23.5 ± 7.8	22.0 ± 7.9	20.3 ± 7.9	19.2 ± 8.3	16.5 ± 8.2	<0.001***
TUGT (−s)	−11.2 ± 4.7	−9.2 ± 2.9	−9.4 ± 2.5	−10.9 ± 3.4	−12.6 ± 5.1	−14.3 ± 6	−16.6 ± 6.1	<0.001***

*Note*: Chi‐square tests were used for categorical variables and Kruskal–Wallis test was used for continuous variables to compare differences between groups.

Abbreviations: ACE‐III, Addenbrooke's cognitive examination‐III; AD1, mild Alzheimer's disease (CDR = 1); AD2, moderate Alzheimer's disease (CDR = 2); AD3, severe Alzheimer's disease (CDR = 3); ALL, all participants; APOEe4, ApoE e4 allele; Aβ, amyloid β‐protein deposition; BMI, body mass index; MAP, mean arterial pressure; MCI, mild cognitive impairment participants; MMSE, mini‐mental state examination; MoCA, Montreal cognitive assessment; NC, cognitively normal participants; PET‐CT, positron emission tomography–computed tomography; SCD, subjective cognitive decline participants; TUGT, timed up and go test.

^a^ Marital status including married (Y) and unmarried/widowed/divorced (N).

^b^ Physical function was obtained by grip strength and TUGT using PCA method.

^c^ Grip strength use both hands for average grip strength.

^d^ The proportion of ALL group refers to the proportion of all PET‐CT participants in all enrolled people, and the proportion of each subgroup refers to the percentage of people in that group as compared to ALL groups.

* 0.01 < *p* < 0.05.

** 0.001 < *p* < 0.01.

*** *p* < 0.001.

### 3.2 Physical function was consistently associated with cognition

**FIGURE 1:**
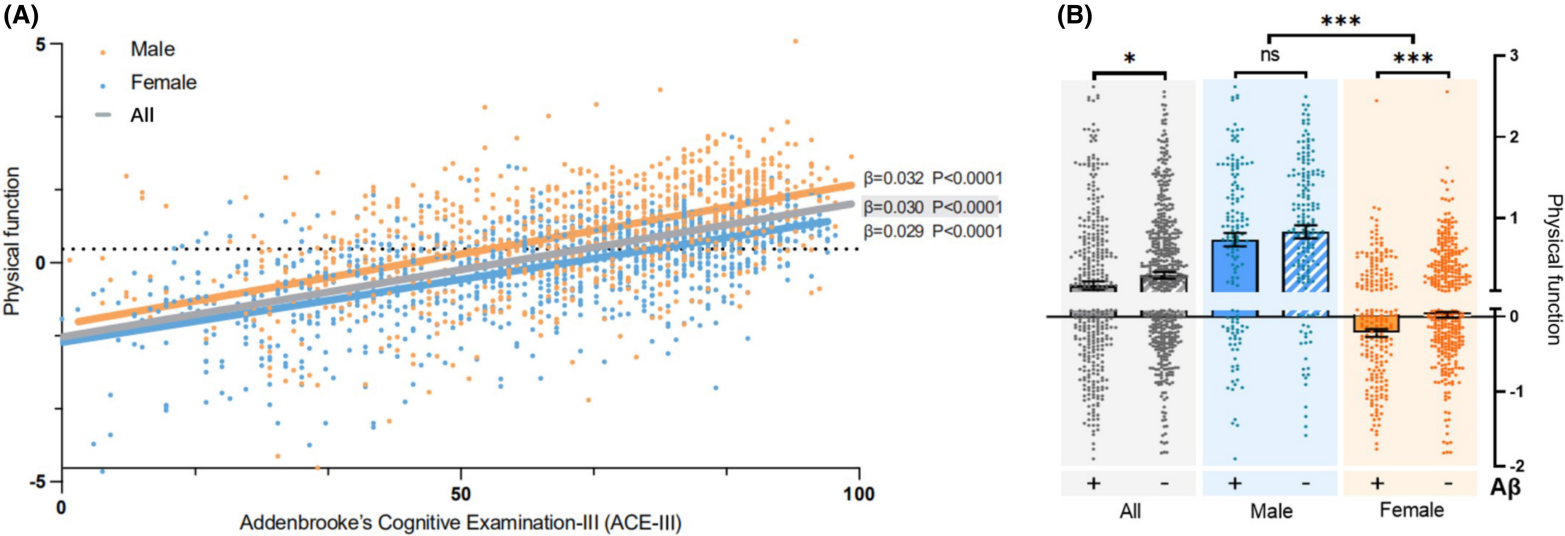
Sex differences in physical function. (A) Scatter plot of ACE score and motor ability. (B) Difference in physical function between Aβ deposition negative and positive groups. Regression curves were fitted using linear regression methods. Kruskal–Wallis test was used for continuous variables to compare differences between groups. *, 0.01 < *p* < 0.05; ***, *p* < 0.001; NS, *p* > 0.05.

Using PCA, grip strength and TUGT scores were dimensionally reduced. The PC1 explaining 65.3% of the variance in the original variables was used as a variable representing physical function. The results of Spearman correlation analysis showed a significant positive correlation (*p* < 0.001) between physical function and ACE‐III scores (Table 2). After controlling for age, sex, and education level as covariates in Model 1, a significant positive correlation (*p* < 0.001) persisted. Model 2, which adjusted for selected variables influencing cognitive function, including age, education level, urinary system diseases, tumors, blood system diseases, and metabolic system diseases (Figure S2), also exhibited a significant positive correlation (*p* < 0.001). Stratification based on sex, age, diagnostic groups, APOE genotypes, and Aβ deposition produced similar results as well. Notably, in the severe AD group, the relationship between physical function and cognition was not significant (*p* = 0.52), indicating that for patients with severe AD, the improvement in physical function no longer exerted an impact on cognitive function. This could be attributed to the fact that in this stage of AD, individuals gradually lose the ability to respond to their environment, carry on a conversation, and eventually control movement.^32^ Here is an inherent sex difference in physical function (*p* < 0.001) (Table S1). However, gender does not have an impact on the relationship between physical activity and cognition (Figure S2). Additionally, in Figure 1A, the overall trends among the total, men, and women groups are consistent.

**TABLE 2:** Correlation between physical function and ACE‐III/MMSE/MoCA‐B score.

	ACE‐III		MMSE		MoCA‐B	
	*R* value (95% CI)	*p* value	*R* value (95% CI)	*p* value	*R* value (95% CI)	*p* value
Crude model	0.54 (0.51–0.57)	<0.001***	0.48 (0.46–0.51)	<0.001***	0.47 (0.44–0.5)	<0.001***
Model 1	0.45 (0.42–0.49)	<0.001***	0.42 (0.39–0.45)	<0.001***	0.40 (0.38–0.43)	<0.001***
Model 2	0.46 (0.42–0.49)	<0.001***	0.42 (0.39–0.45)	<0.001***	0.40 (0.37–0.43)	<0.001***
Sex						
Men	0.52 (0.47–0.57)	<0.001***	0.49 (0.45–0.53)	<0.001***	0.51 (0.46–0.55)	<0.001***
Women	0.57 (0.53–0.61)	<0.001***	0.53 (0.5–0.56)	<0.001***	0.52 (0.49–0.55)	<0.001***
Age						
50–59	0.36 (0.26–0.45)	<0.001***	0.33 (0.26–0.4)	<0.001***	0.25 (0.18–0.33)	<0.001
60–69	0.48 (0.42–0.53)	<0.001***	0.40 (0.35–0.44)	<0.001***	0.38 (0.33–0.43)	<0.001
70–79	0.49 (0.43–0.54)	<0.001***	0.40 (0.35–0.45)	<0.001***	0.38 (0.32–0.43)	<0.001
≥80	0.46 (0.38–0.53)	<0.001***	0.42 (0.35–0.5)	<0.001***	0.40 (0.32–0.48)	<0.001***
Diagnosis						
NC	0.38 (0.30–0.44)	<0.001***	0.32 (0.26–0.37)	<0.001	0.32 (0.26–0.37)	<0.001***
SCD	0.17 (0.04–0.30)	<0.05*	0.12 (0.03–0.22)	0.009**	0.17 (0.08–0.26)	<0.001***
MCI	0.19 (0.10–0.28)	<0.001***	0.16 (0.09–0.23)	<0.001***	0.22 (0.15–0.29)	<0.001***
AD1	0.29 (0.19–0.38)	<0.001***	0.09 (0–0.18)	0.057	0.18 (0.09–0.27)	<0.001***
AD2	0.34 (0.24–0.43)	<0.001***	0.21 (0.12–0.3)	<0.001***	0.25 (0.16–0.33)	<0.001***
AD3	0.05 (−0.11–0.21)	0.52	−0.06 (−0.2–0.07)	0.364	0.14 (−0.1–0.36)	0.243
APOE genotype						
E4 carrier	0.43 (0.28–0.56)	<0.001***	0.39 (0.27–0.49)	<0.001***	0.33 (0.21–0.44)	<0.001***
Non‐E4 carrier	0.38 (0.29–0.46)	<0.001***	0.30 (0.23–0.37)	<0.001***	0.33 (0.26–0.4)	<0.001***
Aβ deposition						
Positive	0.42 (0.29–0.54)	<0.001***	0.37 (0.28–0.45)	<0.001***	0.36 (0.27–0.45)	<0.001***
Negative	0.43 (0.33–0.52)	<0.001***	0.34 (0.26–0.41)	<0.001	0.35 (0.27–0.42)	<0.001***

*Note*: Spearman correlation and Spearman partial correlation were used for correlation analysis. Model 1 corrects for three covariates: age, sex, and years of education. Model 2 corrects for five screened variables that influence the correlation: age, education level, urinary system, tumor, blood system, and metabolic system.

* 0.01 < *p* < 0.05.

** 0.001 < *p* < 0.01.

*** *p* < 0.001.

### 3.3 Sensitivity analysis

Besides ACE‐III score, we performed the associations between physical function and two other scores: the MMSE and Montreal cognitive assessment–basic (MoCA‐B). The results of the Spearman correlation remained consistent with that of ACE‐III (Table 2). We further employed linear regression models to fit the input variables and output variables of Model 1. The results indicated that the models achieved an *R*‐squared value of 0.45 for ACE‐III, 0.37 for MMSE, and 0.42 for MoCA‐B. These results suggest that the combination of physical function with age, sex, and education level can explain approximately 40% of the variability in cognitive scores. We also conducted separate correlation analyses between grip strength, TUGT test scores, and cognitive function scores. Both grip strength (*R* = 0.85, 95% CI: 0.83–0.87, *p* < 0.001) and TUGT test scores (*R* = 0.68, 95% CI: 0.65–0.72, *p* < 0.001) showed a positive correlation with ACE‐III scores. In addition, instead of using PCA for dimensionality reduction, we performed coefficient‐weighted standardization and combined the grip strength and TUGT test scores in another way. As we anticipated, Spearman correlation analysis yielded similar results (*R* = 0.45, 95% CI: 0.37–0.52, *p* < 0.001). Finally, we conducted a validation study involving all participants who underwent PET‐CT scans. The results showed that among Aβ‐positive participants, there was a positive correlation between physical function and cognitive function in non‐CI, MCI, and CI patients (Table S2), suggesting that enhancing physical function is an effective strategy for preventing cognitive decline in individuals who are at risk of developing dementia. Despite the presence of Aβ deposits in the brain, improving physical function remains a beneficial approach to slowing down cognitive decline. Additionally, among Aβ‐negative individuals, who are at low risk of dementia, both those with normal cognition and those with mild cognitive impairment showed a positive correlation between physical function and cognitive function. Collectively, there was a consistent association between physical function and cognitive function.

### 3.4 Physical function was different among populations with different cognitive impairment states

**FIGURE 2:**
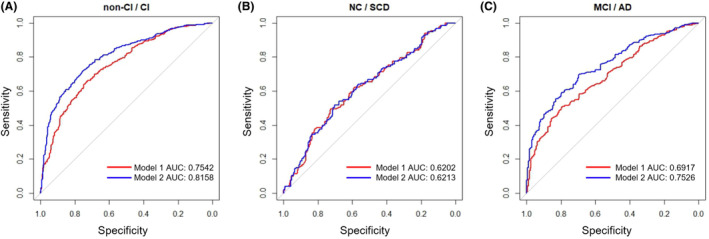
ROC curves for the identification of different phases of AD. (A) Between CI (cognitive impairment, including normal cognition [NC] and subjective cognitive decline [SCD] participants) and non‐CI (including mild cognitive impairment [MCI] and Alzheimer's disease [AD] participants) subgroups. (B) Between NC and SCD subgroups. (C) Between MCI and AD subgroups. ROC curve was plotted using logistic regression model. Model 1 use the crude model with age, sex, and educational level as independent variables. Model 2 uses physical function, age, sex, and educational level as independent variables.

We then categorized participants into two groups: NC participants and those with SCD were grouped in the non‐impaired group (non‐CI), and individuals with MCI and AD were grouped in the impaired group (CI). The combination of age, sex, and educational level was used as the independent variable, while CI and non‐CI were considered as the dependent variables in a logistic regression model for predicting cognitive function. The model achieved an AUC (area under the curve) of 0.759 (Figure 2A), and the AUC for differentiating between NC and SCD participants was 0.591 (Figure 2B), while for distinguishing between MCI and AD participants was 0.728 (Figure 2C). Figure 2A–C also depicts the AUC curves of the logistic regression models for the three classification cases after adding physical function. The AUC values for CI/non‐CI, NC/SCD, and MCI/AD improved to 0.835, 0.635, and 0.777, respectively. These results demonstrated a significant enhancement in the discriminative performance after the inclusion of physical function. Additionally, we separately introduced the two individual variables (TUGT and grip strength) into the model and plotted ROC curves. The results showed that incorporating TUGT or grip strength with covariates increased the AUC values, but the classification performance was not as good as using the combined physical function variable (Figure S3). Together, the physical function holds power in distinguishing cognitive impairment status and effectively differentiating between MCI and AD patients.

### 3.5 Physical function was associated with AD biomarkers

Through analyzing the data from brain PET‐CT scans, we discovered a significant difference in physical function between groups with positive and negative brain Aβ deposition status (*p* < 0.05) (Figure 1B). This difference was more pronounced (*p* < 0.001) in women (Figure 1B), indicating a sex disparity (higher significance in women) in the correlation between physical function and brain Aβ deposition. Using the logistic regression model that was previously employed to discriminate between CI (cognitive impairment) and non‐CI individuals, we determined the best cut‐off value of 0.78 (male) and 0.09 (female) for physical function. Using this cut‐off, we divided the participants into high and low physical function groups.

We further found a significant negative correlation between physical function and whole‐brain imaging SUVr values (*R* = −0.10, 95% CI: −0.17 to −0.03, *p* < 0.01). Subsequently, when stratifying the population, we observed that the correlation was not significant in the men group, but it remained significant in the women group (*R* = ‐0.13, 95% CI: −0.21 to −0.04, *p* < 0.01) (Table 3). Thus, in conjunction with the significant differences in physical function between the Aβ deposition positive and negative groups as mentioned above, we can conclude that physical function was strongly negatively correlated with brain Aβ deposition in women. In a linear regression model that included gender and years of education, the model was significant (*p* = 0.007, *R* ^2^ = 0.02), with physical function exhibiting significance (*β* = −0.05, *p* = 0.008), while age (*p* = 0.69) and education level (*p* = 0.073) were not significant. To determine whether the positive impact of physical function on cognition in the women group was mediated by reduced brain Aβ deposition, we conducted a mediation analysis. The results (Table S3) showed a significant mediating effect (ACME = 0.35, 95% CI: 0.08–0.77, *p* < 0.01), accounting for 0.05 of the total effect (95% CI: 0.01 to 0.10, *p* < 0.01). Furthermore, we performed a correlation analysis of imaging SUVr values in different brain regions (Table 3). We found that the impact of physical function on brain Aβ deposition was mainly evident in the lateral parietal (*R* = −0.13, 95% CI: −0.21 to −0.05, *p* < 0.01), lateral temporal (*R* = −0.14, 95% CI: −0.22 to −0.05, *p* < 0.01), posterior cingulate (*R* = −0.14, 95% CI: −0.22 to −0.06, *p* < 0.001), frontal (*R* = −0.12, 95% CI: −0.21 to −0.04, *p* < 0.01), occipital (*R* = −0.12, 95% CI: −0.20 to −0.03, *p* < 0.01), and precuneus (*R* = −0.14, 95% CI: −0.22 to −0.06, *p* < 0.01) regions.

**TABLE 3:** The correlation between physical function and brain Aβ deposition.

	All (*n* = 836)	Men (*n* = 301)	Women (*n* = 535)
Whole Brain	−0.1 (−0.17 to −0.03)**	0.01 (−0.1 to 0.12)	−0.13 (−0.21 to −0.04)**
Lateral Parietal	−0.11 (−0.18 to −0.04)***	−0.03 (−0.14 to 0.08)	−0.13 (−0.21 to −0.05)**
Lateral Temporal	−0.1 (−0.17 to −0.04)**	0.02 (−0.1 to 0.13)	−0.14 (−0.22 to −0.05)**
Medial Temporal	0.01 (−0.06 to 0.07)	0.08 (−0.03 to 0.19)	0.02 (−0.06 to 0.1)
Posterior Cingulate	−0.11 (−0.18 to −0.04)***	−0.02 (−0.14 to 0.09)	−0.14 (−0.22 to −0.06)***
Amygdala	−0.01 (−0.08 to 0.06)	0.07 (−0.04 to 0.18)	−0.04 (−0.12 to 0.05)
Frontal	−0.1 (−0.16 to −0.03)**	0.02 (−0.09 to 0.14)	−0.12 (−0.21 to −0.04)**
Occipital	−0.1 (−0.16 to −0.03)**	0 (−0.11 to 0.11)	−0.12 (−0.2 to −0.03)**
Precuneus	−0.11 (−0.17 to −0.04)**	−0.03 (−0.14 to 0.09)	−0.14 (−0.22 to −0.06)***
HIPP	0.01 (−0.06 to 0.07)	0.07 (−0.05 to 0.18)	0.05 (−0.04 to 0.13)

*Note*: Spearman correlation was used for correlation analysis.

* 0.01 < *p* < 0.05.

** 0.001 < *p* < 0.01.

*** *p* < 0.001.

**FIGURE 3:**
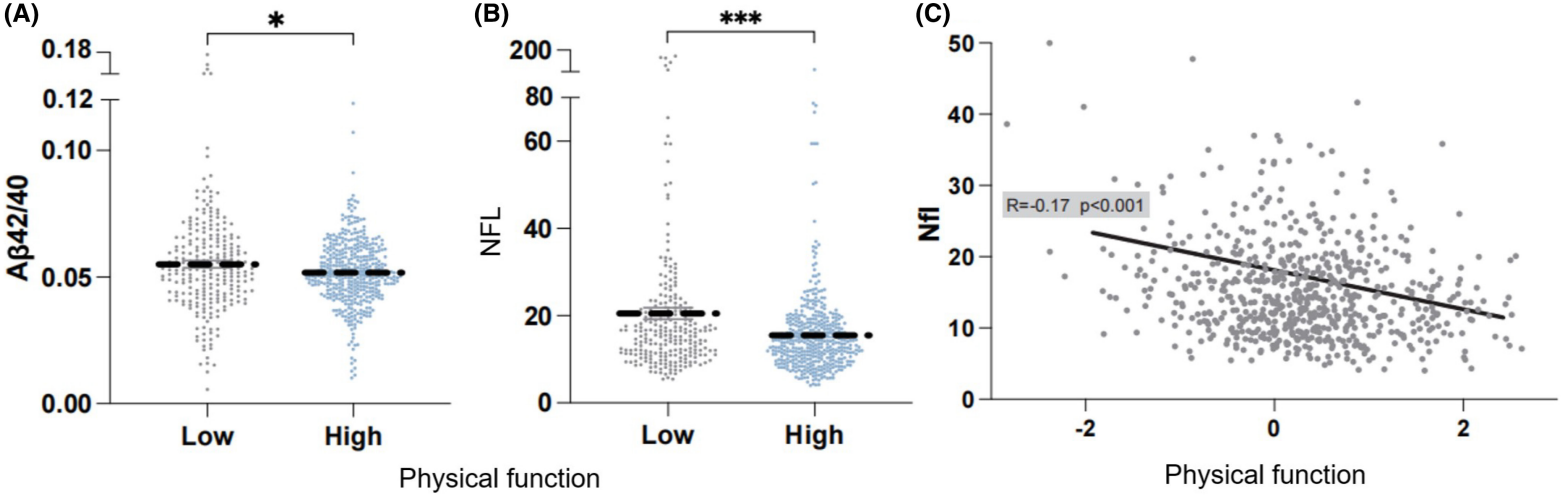
Differences in blood biomarkers between low and high physical function groups. (A) Blood Aβ42/Aβ40 ratio between low motor ability group and high physical function group. (B) Blood Nfl (neurofilament light‐chain) ratio between low physical function group and high motor ability group. (C) Physical function and blood Nfl concentration. Kruskal–Wallis test was used for continuous variables to compare differences between groups. Regression curves were fitted using linear regression methods. *, 0.01 < *p* < 0.05; ***, *p* < 0.001.

We then compared the differences in amyloid, tau, and neurodegeneration (ATN) markers in blood between these two groups and results showed significant differences in the Aβ42/40 ratio (*p* < 0.05) and the concentration of neurofilament light‐chain protein (Nfl) (*p* < 0.001) (Figure 3A,B). Additionally, we performed linear regression to validate the relationship between the ATN markers and physical function and observed a significant negative correlation between physical function and Nfl concentration (*β* = −2.74, *p* < 0.001) (Figure 3C). We also performed stratified analysis to validate the relationship between Nfl and physical function. The negative association between physical function and plasma Nfl remained significant when stratified by gender, APOE genotype, and brain Aβ deposition status (Table S4).

## 4 DISCUSSION

Currently, research on the relationship between exercise and cognition has mostly focused on cognitive changes under exercise interventions^33^, ^34^ or the impact of exercise as a lifestyle habit on cognition.^35^ This is a comprehensive study to investigate the association between physical function and cognitive function. The study highlighted that good physical function acts as a protective factor against cognitive impairment in overall and stratified populations.

Women are nearly twice as likely as men to develop Alzheimer's and live longer than men following an AD diagnosis.^36^ The presence of increased Aβ deposition in women patients likely arises through a combination of factors, including sex chromosomes, hormones, brain structure, and sex and life experiences.^37^, ^38^, ^39^ In our study, we found a significant difference in physical function between the two groups of women participants with different brain Aβ deposition. The positive group showed significantly lower physical function compared to the negative group. Interestingly, this difference was not significant in men. These findings suggest a sex‐specific relationship between physical function and Aβ deposition. We discovered that physical function could influence brain Aβ deposition, and this effect was more prominent in women, where higher physical function was associated with lower brain Aβ deposition. Through mediation analysis, we further confirmed the mediating effect of physical function on cognitive improvement mediated by its impact on brain Aβ deposition. The mediation effect accounting for 6% of the total effect revealed that physical function's influence on cognitive function improvement is not solely mediated by the mechanism of Aβ deposition. There are reports that exercise can improve cognitive decline caused by neurodegenerative diseases through mechanisms such as promoting adult neurogenesis, generating brain‐derived neurotrophic factor, and metabolizing short‐chain fatty acids.^21^ This also explains why higher physical function can lead to better cognitive function in men without altering their brain Aβ deposition status. In the analysis of different brain regions, we found a significant negative correlation between physical function and Aβ deposition in brain regions such as lateral parietal, lateral temporal, posterior cingulate, frontal, occipital, and precuneus. These regions are among the cortical areas where Aβ‐positive patients typically show the most prominent tracer accumulation.^40^ This further confirms the association between the level of physical function and the extent of Aβ deposition in the brain.

Furthermore, we conducted a correlation analysis between physical function and plasma AD biomarkers: A (amyloid‐beta), T (tau), and N (neurofilament light‐chain protein).^41^ We found a significant negative correlation between plasma Nfl levels and physical function. Nfl, as a marker of axonal damage, has received increasing attention in AD diagnosis.^42^ To validate this result, we incorporated ATN simultaneously into a linear regression model and compared its R‐squared value and p‐value with the model using Nfl alone as a predictor. Surprisingly, the significant negative correlation between plasma Nfl levels and physical function remained unchanged, and the contributions of A and T to the model were not significant. This finding supports the negative correlation between physical function and plasma Nfl levels. Previous studies have indicated that acute exercise can lower plasma Nfl levels and increase the flux of neuroprotective pathways.^43^ Therefore, the underlying mechanisms of the relationship between physical function and plasma Nfl levels serve as potential targets for improving cognitive function in AD patients and warrant further exploration.

In sensitivity analysis, we employed coefficient‐weighted standardization to determine and combine the weights of TUGT and grip strength as they relate to cognitive impact. We then validated the reliability of this composite index in regression models. Furthermore, the results of stratified analysis demonstrate that, except for patients with severe AD (individuals lose the ability to respond to their environment, carry on a conversation, and control movement^32^), gender, age, disease progression stage, APOE genotype, and brain Aβ deposition status do not affect the positive correlation between physical function and cognitive function. This underscores the universality of this association. Similar relationships have been observed in studies examining the association between sarcopenia and cognitive function. Research indicates that sarcopenia is a risk factor for cognitive impairment. Meta‐analysis shows that the crude odds ratio (OR) of the association between sarcopenia and cognitive impairment is 2.926 (95% CI: 2.297–3.728). This suggests that improving physical fitness can prevent and mitigate sarcopenia and can have a positive impact on cognitive function.^44^ Notably, after incorporating easily obtainable covariates such as age, sex, BMI, and education level, the composite index demonstrated an *R*‐squared value of 45% for predicting ACE‐III, 37% for MMSE, and 40% for MoCA‐B. These results support the notion that the composite index can effectively explain the variability in cognitive function scores and make accurate predictions. Given the diverse and complicated physical conditions among the elderly, we are devoted to promoting our findings toward clinical translation step by step. Currently, we are recruiting participants for an exercise intervention study that aims to provide exercise prescription recommendations (exercise type, time, frequency, etc.) for individuals with different cognitive and physical function levels, with the help of AI technology and multi‐modal data.

The motoric cognitive risk syndrome (MCR) was proposed in 2013 as a pre‐dementia syndrome that identifies a subgroup of individuals who are at high risk for dementia.^45^ The combination of gait slowness and SCD has been considered as a pre‐dementia syndrome, which is more effective in identifying individuals at high risk of dementia compared to assessing gait slowness or SCD alone.^46^ The confirmed association between physical function and cognitive function in this study, along with the high discriminative ability of physical function for participants with different cognitive statuses, provides a new perspective as a complement and extension to the concept of MCR. Here, we propose physical cognitive impairment syndrome (PCIS) to define a subgroup of individuals characterized by a decline in physical function and a high risk for dementia. Unlike MCR and SCD, which are defined as preclinical stages of dementia, we aim to define PCIS as a pathological state transitioning from MCI to AD. Given the significant negative correlation between physical fitness and Aβ, we hope to identify patients who have already exhibited cognitive impairment accompanied by significantly lower physical function than normal. For individuals over the age of 65 with MCI, physical capability is calculated using the formula (average grip strength × 0.084 + TUG test × 0.152–0.135). Men with a score below 0.78 and women with a score below 0.09 are classified as having PCIS. Non‐PCIS patients do not exhibit the clinical phenotype of decreased physical capability, whereas PCIS patients will show improvements in physical capability and a slowdown in cognitive decline following active interventions. This diagnosis criteria will help us implement exercise interventions to slow down the progression to AD. The specific evaluation criteria and corresponding validation will be refined in our subsequent research.

Our study has certain limitations. Firstly, our cohort primarily consists of older adults from the Yangtze River Delta region, especially Shanghai. The limited geographical coverage may restrict the generalizability of our findings, and further validation is needed in a more diverse population including different ethnicities, regions, language habits, and lifestyles. Secondly, not all participants underwent all the examinations, resulting in significant missing data and participants. The limited geographical coverage and many other covariates (e.g., comorbidity, medication, exercise habit, and test mode) may restrict the generalizability of our findings, and further validation is needed in diverse population. Thirdly, as an observational study, there may be recall biases during the questionnaire process, and some accompanying individuals may not be the actual caregivers of the patients. Another limitation is that while we have identified certain correlational findings, we have not delved deeply into the underlying mechanisms. Future research should aim to gain a more comprehensive understanding of these mechanisms to better explain the phenomena observed.

## 5 CONCLUSION

In conclusion, this study underscores a positive association between physical function and cognitive function, indicating that physical function partly reflects an individual's cognitive function. These findings provide new insights into the diagnosis and mitigation of AD. Additionally, the relationship among brain Aβ deposition, plasma Nfl levels, and physical function was identified, pointing toward future directions for mechanistic research. Early diagnosis and identification of AD are crucial for disease intervention, reducing disease burden, and improving the quality of life for older adults.

## CONFLICT OF INTEREST STATEMENT

The authors declare that they have no competing interests.

## Supporting information


Table S1.
Table S2.
Table S3.
Table S4.
Table S5.
Figure S1.
Figure S2.
Figure S3.

## Data Availability

The data that support the findings of this study are available from the corresponding author upon reasonable request.
