# Supplementary material for: Physical function is associated with cognitive status, brain amyloid‐beta deposition, and blood biomarkers in Chinese Han population
Source: CNS Neurosci Ther. 2024 Aug 18;30(8):e14921. doi: 10.1111/cns.14921 (PMC11330986; doi:10.1111/cns.14921)
Supplement: Supplementary file 1 — Table S1. Table S2. Table S3. Table S4. Table S5. Figure S1. Figure S2. Figure S3. [file CNS-30-e14921-s001.docx]

**Supplementary material**

Table.S1 Demographic and clinical characteristics of study participants (grouped by gender, n=4189)

|  | **ALL(n=4189)** | **Men(n=1597)** | **Women(n=2592)** | **p value** |  |
| --- | --- | --- | --- | --- | --- |
| Age(yr) | 67.9 ± 11.5 | 69.4 ± 11.5 | 67 ± 11.3 | <0.001 | *** |
| Education(yr) | 11.3 ± 4.2 | 12.0 ± 4.1 | 10.8 ± 4.2 | <0.001 | *** |
| Marriage(Y/N)^1^ | 2160/351 | 782/68 | 1378/283 | <0.001 | *** |
| BMI(kg/m2) | 23.3 ± 3.3 | 23.7 ± 3.3 | 23 ± 3.3 | <0.001 | *** |
| Wasit(cm) | 85.3 ± 9.6 | 89.5 ± 8.9 | 83.1 ± 9.3 | <0.001 | *** |
| MAP(mmHg) | 96.6 ± 11.9 | 98.4 ± 11.7 | 95.6 ± 11.8 | 0.008 | ** |
| Smoke(Y/N) | 564/3307 | 527/933 | 37/2374 | <0.001 | *** |
| Drink(Y/N) | 670/3196 | 536/924 | 134/2272 | <0.001 | *** |
| Family history(Y/N) | 1561/1134 | 555/394 | 1006/740 | 0.694 |  |
| Medication history(Y/N) | 472/3717 | 193/1404 | 279/2313 | 0.207 |  |
| PET-CT^4^ | n=1048, 25% | n=402, 38% | n=646, 62% | 0.908 |  |
| Aβ(+/-) | 426/622 | 177/225 | 249/397 | 0.090 | . |
| APOEe4(+/-) | 305/788 | 118/300 | 187/488 | 0.905 |  |
| MMSE | 22.9 ± 7 | 22.7 ± 6.8 | 23 ± 7.1 | 0.103 |  |
| MoCA-B | 19.8 ± 7.1 | 19.3 ± 6.9 | 20.1 ± 7.1 | 0.001 | ** |
| ACE-Ⅲ | 61.4 ± 21.8 | 62.5 ± 21.1 | 60.8 ± 22.3 | 0.048 | * |
| Physical function^2^ | 0.0 ± 1.1 | 0.4 ± 1.2 | -0.3 ± 1 | <0.001 | *** |
| Grip strength(kg)^3^ | 22.0 ± 8.5 | 27.9 ± 9 | 18.5 ± 5.8 | <0.001 | *** |
| TUGT(-s) | -11.2 ± 4.7 | -11.5 ± 4.6 | -11.1 ± 4.7 | 0.004 | ** |

**Note**: Chi-square tests were used for categorical variables and Kruskal-Wallis was used for continuous variables to compare differences between groups. ., 0.1<p<0.05; *, 0.05<p<0.01; ***, p<0.001; 1, Marital status including married (Y) and unmarried/widowed/divorced (N); 2, Physical function was obtained by grip strength and TUGT using PCA method; 3, Grip strength use both hands for average grip strength. 4, The proportion of ALL group refers to the proportion of all PET-CT participants in all enrolled people, the proportion of each subgroups refers to the percentage of people in that group as compared to ALL groups.

**Abbreviations**: ALL, all participants; BMI, Body mass index; MAP, Mean arterial pressure; PET-CT, positron emission tomography computed tomography; Aβ, amyloid β-protein deposition; APOEe4, ApoE e4 allele; MMSE, mini-mental state examination; MoCA, Montreal Cognitive Assessment; ACE-III, AddenBrooke’s cognitive examination-III; TUGT, Timed Up and Go test.

Table S2 Correlation between physical function and cognitive function in Aβ deposit subgroup

|  |  | **R value(95%CI)** | **P value** |  |
| --- | --- | --- | --- | --- |
| **Aβ deposit** | |  |  |  |
|  | non-CI(NC+SCD) | 0.26(0.01~0.48) | 0.040 | * |
|  | MCI | 0.36(0.07~0.59) | 0.018 | * |
|  | CI(AD) | 0.28(0.05~0.49) | 0.021 | * |
| **non-Aβ deposit** | |  |  |  |
|  | non-CI(NC+SCD) | 0.43(0.32~0.54) | <0.001 | * |
|  | MCI | 0.30(0.06~0.50) | 0.016 | * |

**Note**: Spearman correlation was used for correlation analysis. 0.1<p<0.05; *, 0.05<p<0.01; ***, p<0.001

Table S3 Mediation effect analysis

|  | **Estimate(95%CI)** | **p-value** |  |
| --- | --- | --- | --- |
| ACME | 0.35(0.08~0.77) | <0.01 | ** |
| ADE | 7.52(5.82~9.22) | <0.001 | *** |
| Total Effect | 7.88(6.20~9.64) | <0.001 | *** |
| Prop.Mediated | 0.05(0.01~0.10) | <0.01 | ** |

**Abbreviations**: ACME, average causal mediation effects; ADE, average direct effects.

Table.S4 Correlation between physical function and plasma Nfl

|  |  | **R value** | **P value** |
| --- | --- | --- | --- |
| crude model | | -0.17 | <0.001 |
| Sex | |  |  |
|  | male | -0.29 | <0.001 |
|  | female | -0.19 | <0.001 |
| APOE genotype | |  |  |
|  | E4-carrier | -0.23 | 0.002 |
|  | non-E4-carrier | -0.17 | <0.001 |
| Aβ deposition | |  |  |
|  | positive | -0.17 | 0.001 |
|  | negative | -0.18 | 0.003 |

**Note:** Spearman correlation was used for correlation analysis.

Table.S5 Shapiro-Wilk test result of all continuous variable

|  | **W value** | **P value** |
| --- | --- | --- |
| Age | 0.964 | <0.001 |
| Educational level | 0.971 | <0.001 |
| Height | 0.992 | <0.001 |
| Weight | 0.985 | <0.001 |
| BMI | 0.975 | <0.001 |
| Waist | 0.997 | 0.001 |
| MAP | 0.988 | <0.001 |
| Grip strength | 0.969 | <0.001 |
| TUGT | 0.773 | <0.001 |
| Physical function | 0.956 | <0.001 |
| MMSE | 0.855 | <0.001 |
| MoCA | 0.937 | <0.001 |
| ACE-Ⅲ | 0.950 | <0.001 |
| Whole Brain SUVr | 0.531 | <0.001 |
| LateralParietal SUVr | 0.489 | <0.001 |
| LateralTemporal SUVr | 0.594 | <0.001 |
| MedialTemporal SUVr | 0.979 | <0.001 |
| PosteriorCingulate SUVr | 0.747 | <0.001 |
| Amygdala SUVr | 0.985 | <0.001 |
| Frontal SUVr | 0.527 | <0.001 |
| Occipital SUVr | 0.476 | <0.001 |
| Precuneus SUVr | 0.566 | <0.001 |
| HIPP SUVr | 0.980 | <0.001 |
| Plasma Aβ40 | 0.977 | <0.001 |
| Plasma Aβ42 | 0.988 | <0.001 |
| Plasma T-Tau | 0.754 | <0.001 |
| Plasma P-Tau181 | 0.302 | <0.001 |
| Plasma Aβ42/40 | 0.878 | <0.001 |
| Plasma Nfl | 0.553 | <0.001 |
| Plasma P-Tau181/Aβ42 | 0.015 | <0.001 |

**Abbreviations**: BMI, Body mass index; MAP, Mean arterial pressure; TUGT, Timed Up and Go test; MMSE, mini-mental state examination; MoCA, Montreal Cognitive Assessment; ACE-III, AddenBrooke’s cognitive examination-III.

Fig. S1 Formation of the study population.


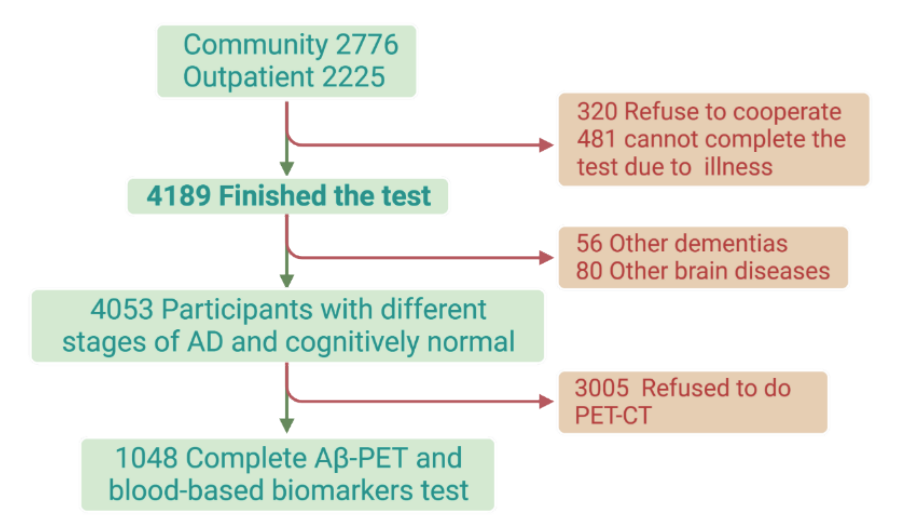


Fig. S2 Confounders-filtered forest map

**Note**: The β coefficient and 95%CI were obtained by subtracting the coefficients of the two models from Bootstrap1000 times. Variables with a 95% confidence interval spanning over the value of 0 are identified as confounding factors affecting the relationship between physical function and cognition.

**Abbreviations**: APOE, ApoE genotype; Drug, Medication history; famhis, Family history; S1~S9, Respiratory system, Cardiovascular system, Endocrine system, digestive system, Urinary system, Skeletal system, nervous system, Tumor, blood system, Metabolic system disease history; MAP, Mean arterial pressure; BMI, Body mass index; hand, Dominant hand; Employ, Employment situation; Edu, educational level

Fig. S3 ROC curves for the identification of different phases of AD (grip strength and TUGT).

**
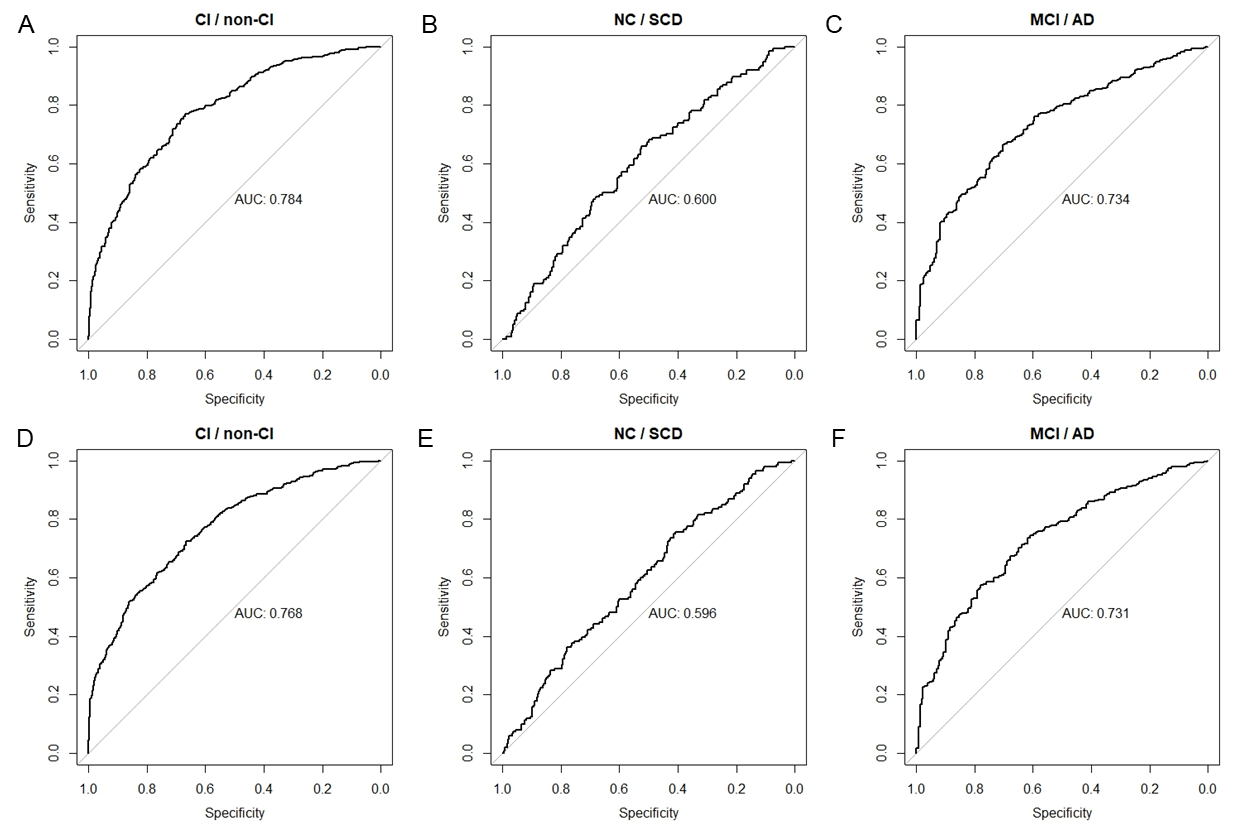
**

ROC curves for the identification of different phases of AD (A) In the CI (cognitive impairment, including NC and SCD participant) and non-CI (including MCI and AD participant) subgroup. (B) In the NC and SCD subgroup. (C) In the MCI and AD subgroup. (D) In the CI and non-CI subgroup. (E) In the NC and SCD subgroup. (F) In the MCI and AD subgroup.

**Note**: ROC curve was plotted using logistic regression model. (A), (B) and (C) plot use the model with TUGT score age, sex and educational level. (D), (E) and (F) plot use the model with grip strength, sex, age and educational level.
